# Development of a hospital-based patient-reported outcome framework for lung cancer patients: a study protocol

**DOI:** 10.1186/s12955-017-0837-z

**Published:** 2018-01-11

**Authors:** Natasha Moloczij, Karla Gough, Benjamin Solomon, David Ball, Linda Mileshkin, Mary Duffy, Mei Krishnasamy

**Affiliations:** 1 Peter MacCallum Cancer Centre, Locked Bag 1, A’Beckett Street, Melbourne, VIC 8006 Australia; 2 Department of Nursing, School of Health Sciences, University of Melbourne, Melbourne, Australia; 3 Victorian Comprehensive Cancer Centre, Melbourne, Australia; 4 Centre for Cancer Research, University of Melbourne, Melbourne, Australia

**Keywords:** Oncology, Patient-reported outcomes, Lung cancer, Quality of care, Feasibility

## Abstract

**Background:**

Patient-reported outcome (PRO) data is central to the delivery of quality health care. Establishing sustainable, reliable and cost-efficient methods for routine collection and integration of PRO data into health information systems is challenging. This protocol paper describes the design and structure of a study to develop and pilot test a PRO framework to systematically and longitudinally collect PRO data from a cohort of lung cancer patients at a comprehensive cancer centre in Australia.

**Methods:**

Best-practice guidelines for developing registries aimed at collecting PROs informed the development of this PRO framework. Framework components included: achieving consensus on determining the purpose of the framework, the PRO measures to be included, the data collection time points and collection methods (electronic and paper), establishing processes to safeguard the quality of the data collected and to link the PRO framework to an existing hospital-based lung cancer clinical registry. Lung cancer patients will be invited to give feedback on the PRO measures (PROMs) chosen and the data collection time points and methods. Implementation of the framework will be piloted for 12 months. Then a mixed-methods approach used to explore patient and multidisciplinary perspectives on the feasibility of implementing the framework and linking it to the lung cancer clinical registry, its clinical utility, perceptions of data collection burden, and preliminary assessment of resource costs to integrate, implement and sustain the PRO framework. The PRO data set will include: a quality of life questionnaire (EORTC-QLQ-C30) and the EORTC lung cancer specific module (QLQC-LC-13). These will be collected pre-treatment (baseline), 2, 6 and 12 months post-baseline. Also, four social isolation questions (PROMIS) will be collected at baseline.

**Discussion:**

Identifying and deciding on the overall purpose, clinical utility of data and which PROs to collect from patients requires careful consideration. Our study will explore how PRO data collection processes that link to a clinical data set can be developed and integrated; how PRO systems that are easy for patients to complete and professionals to use in practice can be achieved, and will provide indicative costs of developing and integrating a longitudinal PRO framework into routine hospital data collection systems.

**Trial registration:**

This study is not a clinical trial and is therefore not registered in any trial registry. However, it has received human research ethics approval (LNR/16/PMCC/45).

## Background

Measuring and integrating patient reported outcomes (PROs) are increasingly recognised as central to the delivery of quality health care [1–3]. Crucially, outcomes that matter to patients such as time to return to work, functional performance, impact of side effects of treatment on daily functioning, and emotional wellbeing are commonly absent from hospital reporting metrics [2]. When routinely combined with clinical data, PROs provide important information that have potential to translate to meaningful service improvements [1]. However, integration of routinely collected PRO data into hospital-based health information systems is challenging [4]. In particular, there is little understanding of the resources required to develop, implement, utilise and sustain a clinically relevant PRO framework [5]. The level of investment is determined by how many variables are to be collected, the complexity of data collection processes (for example, how many times patients are followed up; what happens if data are missing; hard copy or electronic systems), integration of quality assurance data collection processes and reporting requirements [6]. Therefore, it is vital to pilot and evaluate the feasibility of collecting PRO data from patient, clinician and system perspectives. Establishing the clinical utility of the data gathered, and the cost of integrating a PRO framework into a health care setting are essential first steps before committing to system-wide PRO infrastructure [7].

### Study aims

The aims of our study are to:

1. Develop a PRO framework to systematically collect longitudinal PRO data and can be linked to a lung cancer clinical registry at a comprehensive cancer centre in Australia, and
2. To pilot test and evaluate the feasibility and clinical utility of the PRO framework.

If proven to be feasible, clinically meaningful and sustainable, data from the PRO framework will be used to examine clinical utility and PROs, inform models of care and generate benchmarking data for the lung service at the cancer centre. Furthermore, lessons from the pilot study will inform expansion of the PRO framework into other tumour services and patient groups across the organisation. The principals and steps to develop the PRO framework, and consequently the protocol, were informed by best-practice clinical quality registry and PRO registry guidelines [6–8].

## Methods

**Fig. 1:**
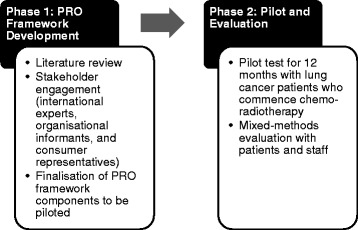
Study flow diagram

To meet the research aims, the protocol has been developed as a two-phase initiative: 1) development of a PRO framework for collecting PRO data; and 2) a 12-month feasibility study (see Fig. 1). The PRO framework was developed using a combination of best-practice guidelines for establishing registries for PRO data and a consensus-based approach with key stakeholders [6]. The development phase occurred prior to submission of the protocol to Human Research Ethics Committee (HREC) but was included in the submitted protocol to ensure critical scientific and ethical review. This study protocol has been approved by the local HREC (LNR/16/PMCC/45, protocol version 5).

### PRO framework development

**Fig. 2:**
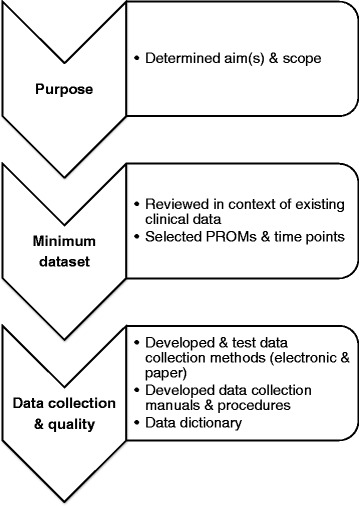
Key components of patient-reported outcome framework

Phase 1 focused on establishing the PRO framework aims, scope, infrastructure and prospective data collection methods. Consensus about these essential components was achieved through consultations with key stakeholders including consumer representatives, members of the multi-disciplinary team members within the organisation, international cancer experts, and drawing on best-practice guidelines for the development of registries for evaluating patient outcomes [2, 5, 6] and the Framework for Australian Clinical Quality Registries [8]. During the consensus process several lung cancer PRO frameworks were considered, such as the International Consortium for Health Outcomes Measurement lung cancer data set [9], the UK National Lung Cancer Audit [10], and the Victoria Lung Cancer Registry [11]. However, there were translational issues identified when considering these frameworks for our setting. For instance, the specific patient group for whom the data set had been developed and implemented with (e.g. early stage lung cancer patients eligible for surgery); differing clinical processes, (e.g. the ability to collect baseline measures prior to different treatments); local resource infrastructure (e.g. electronic medical record systems); and specific areas of PRO clinical interest or intent (e.g. bench-marking data versus real-time clinical decision-making). Thus, existing frameworks were not fully applicable to our setting and so a best-practice approach was applied [5]. Paying attention to this level of clarity around intent, purpose, and local context was an early an important lesson and is detailed below. Figure 2 shows three key components of the PRO framework that were developed in the first phase of the study that informed which PRO measures (PROMs) to include and what data collection processes should be piloted in the second phase.

### Establishing the purpose of the PRO framework

The first step in the development process focused on establishing agreement about the purpose of the PRO data, what PRO data to collect, detailing a data analysis plan, examining potential for data utility and sustainability of the framework [6]. There was unanimous agreement that the framework should enable integration of clinical and PRO data sets - a ‘bench to home’ approach and be used to optimise clinical outcomes and improve patient care. As the lung cancer service already collects prospective clinical data as part of a Thoracic Malignancies Cohort (TMC) study, it was agreed that the PRO framework should link with and leverage clinical data already collected through the TMC study.

The study team determined that for feasibility purposes, the PRO framework should pilot test data collection processes with a clearly defined subset of TMC lung cancer patients. As such, the patient inclusion criteria set for the 12 month pilot study are: patients diagnosed with lung cancer, English speaking, aged 18 years and over, have consented to the TMC study, and are about to commence chemo-radiation treatment.

### Defining the minimum dataset

**Fig. 3:**
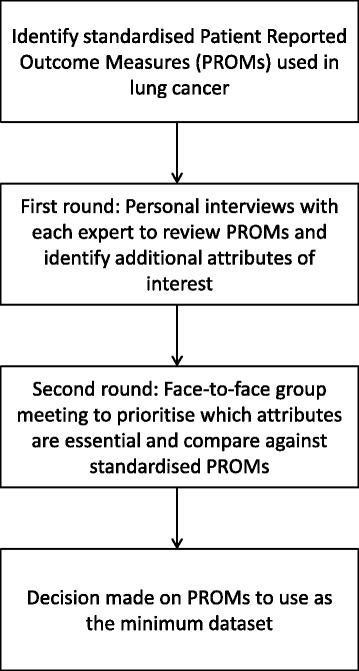
Process used to identify and select PROMs minimum dataset

Another key step in the development process was to decide how much and what type of PRO data to collect from patients without this being burdensome [7]. Careful consideration was given to the choice of standardised PROMs to be included. As the decision had been made to align PRO data with the TMC data set available in order to optimise clinical and patient outcomes, clinicians were consulted about the minimum PRO dataset to be tested in the pilot study. Critically however, patient feedback on the relevance and acceptability of the PROMs will inform the final data set prior to integration into usual care. This is a key objective of the pilot study. The process to determine which PROMs to pilot included a two-phase reactive Delphi [12]. Phase one involved a search of the literature to identify what standardised lung cancer PROMs were available. Although generic PROMs are available, disease-specific PROMs were agreed to be of greater clinical utility with regard to identifying disease, treatment-related symptoms and side-effects amenable to clinical intervention. Phase two involved consultation with an expert, multi-disciplinary reference group made up of medical and radiation oncologists, specialist oncology nurses and an oncology dietician to guide the PROMs selection process. An overview of the process undertaken to reach consensus is presented in Fig. 3.

The aim of the first round of the Delphi was to generate a list of PRO domains to be covered by the PROMs minimum dataset. Interviews were conducted with each expert individually and discussion prompted by presentation of results from a recently published review of PROMs [13]. Each expert was asked to consider how lung cancer patients’ lives might be impacted by lung cancer and the chemo-radiation treatment. Items from each of the PROMs under consideration (and made available to the experts during the interviews) were reviewed or discussed. Each time an expert identified a specific PROMs item as being of relevance, it was given a score of one. All of the response scores were then totalled.

The results were anonymised, summarised and presented to the experts in a group meeting. During the meeting, the highest scoring items as identified by the experts were considered alongside two standardised PROMs that included most of the attributes identified by the expert participants during the one-on-one interviews. In particular, consideration about the clinical utility, acceptability and relevance of the two PROMs were discussed. Topics discussed were the potential for integration with clinical data collected as part of the TMC, availability in languages other than English, the number of questions – data burden for participants, and the psychometric properties. This process resulted in consensus among the expert group, and a decision that the minimum dataset should include: the EORTC QLQ-C30 and QLQ-LC13 [14]. Additionally, the four item Patient-Reported Outcome Measurement Information System (PROMIS®) Social Isolation v2.0 –Short Form 4a [15], and patient weight should be collected at baseline only (that is, prior to treatment commencement). The PROMIS® Social Isolation v2.0 –Short Form was considered important for inclusion because of the associated risk of increased mortality [16, 17]. Weight loss was considered important due to its association with functional decline and frailty [18, 19] and its prognostic value [20].

A recognised challenge when collecting PROs is to minimise missing data [7, 21]. There are many different design strategies to reduce missing data [21], with one of the key being optimising timing of PRO data collection. Clinicians identified when patients were most likely to experience side effects from chemo-radiation therapy, what were clinically meaningful periods that may impact data reported (for example, scans, re-staging, end of treatment), and prognosis. This information was balanced with a key focus on minimising burden for patients from completing PROMs at multiple time points. Consequently, the following time points were selected: baseline (prior to treatment commencement), as well as 2, 6 and 12 months post-baseline.

### Data collection and data quality

The method in which PRO data was to be collected from patients also required careful deliberation. Electronic data collection has many benefits and is now considered the preferred method of collecting PRO data, but there are recognised limitations [7]. An essential requirement for electronic data collection is accessibility and user comfort with technology [7]. Given that this is a pilot study to test the feasibility of collecting PROs, the decision was made to collect data by both paper and electronic methods to help inform decisions with regard to lung service wide implementation of the PRO framework, should the results of the pilot study be positive.

Therefore, during the consent process for the pilot study, participants will be given the option of completing their follow-up PROMs on paper, or online using REDCap (Research Electronic Data Capture) [22]. The baseline measures will be completed face-to-face or electronically with the research officer. REDCap is a secure, web-based application designed to support data capture for research studies, providing: 1) an intuitive interface for validated data entry; 2) functionality to track and send reminders to increase data completion; 3) audit trails for data manipulation and export procedures; 4) automated export procedures for seamless data downloads to common statistical packages; and 5) procedures for importing data from external sources. Participants who opt to complete PROMs electronically will provide their email address and automatic email invitations will be set up in the REDCap system to send links and reminders to complete follow-up PROMs at 2, 6 and 12 months post-baseline. Data collected using REDCap will be stored in a secure REDCap web-based database, and later linked to a Microsoft Access database that will store data collected from hard-copy PROMs. Using REDCap will enable setting up of mandatory fields to reduce missing data as incomplete questions will prevent submission of a completed PROM set at each time point.

Once the data collection processes were established, four patients who had previously been treated with chemo-radiation for lung cancer were invited to join the study as consumer representatives on the study Consumer Working Group and study Steering Committee. Involving consumers in the study governance structures as well as study design and process issues will ensure ongoing consumer engagement and influence across all aspects of the PRO framework study. The consumers were identified through the lung service as recently receiving chemo-radiation treatment. Consumer representatives were asked to provide feedback on the PROMs chosen and the data collection methods. Consumers were briefed on the aims and objectives of the study, consented to take part as expert advisors and were given copies of the PROMS to review and complete. Later, they were contacted by telephone by the research officer to give feedback about the relevance of the PROMs items, and asked for their views about hard copy or electronic PROMs completion. Consumer feedback and general observations about the PROMs were recorded as field notes by the research officer. Feedback from the consumer representatives were used to refine the PRO framework processes and protocol design details ahead of phase two.

To ensure data quality, a data management plan and data dictionary was drafted. These documents include: description of data collection processes to ensure standardisation of data and administration completion; quality assurance activities (such as random checks on the data entered) to monitor the completeness and accuracy of manually entered data, documentation of data flows (such as workflow diagrams of how data is synced between REDCap and Microsoft Access databases) from various sources and data security. Throughout phase two, the data management plan and data dictionary will continually be updated and refined.

### Pilot and evaluation

**Fig. 4:**
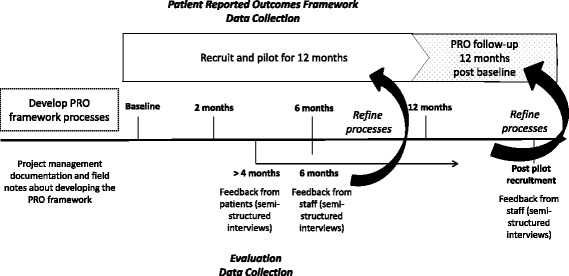
Pilot and evaluation design overview

The aim of the second phase of the study is to pilot the PRO framework for 12 months and to assess feasibility, clinical utility and sustainability of collecting PROs from a cohort of patients with lung cancer. A mixed-methods, process and impact evaluation design will be used to refine implementation processes and establish feasibility of implementing and integrating the PRO framework within a clinical setting [23]. Data collected to evaluate feasibility will include: retention rates, level of completeness of PROMs data, evaluation questions to be directed at identifying the relevance and acceptability of the PROMs used, interview feedback from patients and staff about their experience of completing the PROMs and their attempts at using the PRO data in clinical contexts. Figure 4 outlines the design of the framework and evaluation.

### Sample size

The sample size set for the pilot study is pragmatic and determined by the number of lung cancer patients referred to the cancer centre for chemo-radiation over a 12 month period. Based on 2014–2015 data, approximately 30–60 patients are anticipated to be eligible for participation. Given that patients will be asked to complete a consent form (i.e., an opt-in consent process), and inability at this stage of the study to include patients whose preferred language is not English, a final convenience sample of between 20 and 30 patients was set as the target for recruitment.

### Patient recruitment

Potentially eligible patients will be identified either via a screening list or by members of the lung cancer service. The research officer will identify patients’ treatment start date and approach them to consider participation. Due to screening processes and required medical investigations prior to treatment commencement, it is estimated that there will be up to a two-week window to allow adequate time for patients to consider their participation. A verbal explanation, either in person or by telephone, and a Participant Information Sheet and Consent Form will be given to patients by the researcher officer. Prior to their first chemo-radiation treatment, patients who consent to participate and complete the baseline measures, will choose how they would like to receive and complete follow-up PROs questionnaires.

### Data collection

#### Patient-reported outcome measures

The PRO questionnaire will consist of 48 questions before treatment commencement (baseline), and 44 questions at each of the follow-up time points (2, 6 and 12 months post-baseline). It is estimated that it will take between 10 and 20 min to complete the PRO suite. The EORTC QLQ-C30 is a validated 30-item PROM incorporating five functional scales (physical, role, cognitive, emotional, and social functioning), three symptom scales (fatigue, pain, and nausea/vomiting), a global health status scale (GHS), and six single items assessing dyspnoea, sleep disturbance, appetite loss, constipation, diarrhoea, and financial impact [14]. The EORTC Lung Cancer module is a validated PROM comprising 13 questions. The module incorporates one multi-item scale to assess dyspnoea, and a series of single items assessing pain, coughing, sore mouth, dysphagia, peripheral neuropathy, alopecia, and haemoptysis [24].

The PROMIS® Social Isolation-Short Form is a validated PROM which assesses perceptions of being avoided, excluded, detached, disconnected from, or unknown by, others [25]. The PROMIS® Network short forms comprise items which were first evaluated using classical test theory indices. Unidimensionality was confirmed via confirmatory factor analytic techniques [15]; and then item response theory modelling and expert review was used to identify items measuring the entire spectrum of the construct targeted by each scale. All relevant short forms are standardised, accurate, and efficient self-report measures. A self-reported measure of weight has also been included into the PRO questionnaire.

#### Evaluation data

At the end of the PRO questionnaire, additional closed and open-ended evaluation questions will be included to allow patients to provide feedback on their experience of completing the PRO suite, such as perception of time taken to complete the questionnaire, types of questions asked and relevance to personal circumstances. Project management field notes will record the amount of time and resources taken to develop, integrate and run the PROs framework, establish set up and the ongoing running costs. This will include but not be limited to: protocol development, resources for recruitment, data collection, data entry, cleaning, analysis, reporting, IT infrastructure, collaborating with stakeholders and administrative tasks.

Semi-structured interviews will be undertaken with patients and clinical staff to identify if and what possible refinements to the PRO framework could be made to enable long-term sustainability and acceptability of the PRO framework. Up to ten patients will be interviewed at least four months after they have consented to take part in the pilot study. Patients will indicate on the consent form, at entry to the study, if they are interested in taking part in an interview at a later date. Purposive sampling will be used to ensure variation in responses. The following variables will guide recruitment to the evaluation interviews; completion of the PROMs across all time points, reported experience of completing the PROMs (that is, feedback via survey questions) and/or questionnaire responses (for example, low and high symptom scores). Semi-structured, open-ended questions will focus on thoughts about frequency of completing the PROMs, the method of follow-up, types of questions asked, and what else could be included or removed.

Clinical staff involved in the PRO framework development and implementation process will also be invited to take part in semi-structured interviews. Purposive sampling will be used to obtain a variety of clinical perspectives about the pilot implementation processes and data utility. A total of six staff will be interviewed at two time points, six months into, and on completion of the pilot study. Semi-structured questions will explore their perceptions about the impact of the PRO framework on their workload, clinical consultations/decision-making, how processes could be improved, and the essential infrastructure factors required to collect and use PRO data in a sustainable and clinically relevant way.

### Data management and analyses

All PRO data will be used for the evaluation. Data analysis will explore the method of completion at each time point, identification of missing data, and acceptability or burden of PROMs completion. Descriptive statistics (counts and percentages; means and standard deviations or medians and interquartile ranges, as appropriate) will be used to summarise feasibility and sustainability data, as well as patient characteristics and PRO data. Firth’s bias reduced logistic regression will be used to model the probability of consenting to the study (versus declining) and having a missing form(s) (versus having complete data) [26]. This will be done separately for each predictor that will include but not be limited to age, sex, stage and ECOG. After inspection of the data, the appropriateness of the suggested analysis methods will be reviewed, and revised if necessary. Data will be analysed using R (reference index version 3.2.0 or higher) [27]. Regression analysis will be performed using the “logistf” package [28].

All interviews will be recorded and transcribed. Qualitative software, NVivo10 [29], will be used to manage data. A thematic, descriptive approach [30] will be used to ascertain important findings about collecting and/or completing PROMs, the reminder processes, data integration with current hospital information systems and to explore the clinical utility of PRO data. Staff interview and patient feedback data will be analysed to explore opportunity for clinical (care and treatment) improvements. For example, to consider feasible and appropriate follow-up processes where data indicate high or concerning individual PRO scores (system and individual level clinical utility), and exploration of trends of symptoms and levels of functioning across PRO time points, to consider clinical utility at a cohort level.

## Discussion

This protocol paper describes the comprehensive process undertaken and underway to develop, pilot and evaluate a framework to routinely collect longitudinal PRO data in a cancer setting. For PRO data to be relevant, appropriate and useful in a clinical setting requires collaboration and input with a variety of key stakeholders. One of the most important steps is establishing an approach to select a set of clinically relevant PROMs that can be used to inform health care improvements while not overly burdening patients. There is high quality evidence in the literature about the benefits of collecting and using PROs, in particular for treatment monitoring, detection of symptoms, and improved patient-clinician communication and patient satisfaction [31]. However, there are many considerations that need attention to enable long-term, quality collection and use of PRO data within routine clinical settings [5, 32]. These include perceptions about the usefulness and appropriateness of the types of questions asked, difficulty incorporating PRO data into clinical practice, lack of familiarity of how to interpret or respond to the data, reporting for quality improvement purposes, logistical and resource constrains [5, 32]. Our study will explore how PRO data collection processes that link to a clinical data set can be developed and integrated; how PRO systems that are easy for patients to complete and professionals to use in practice can be achieved, and will provide indicative costs of developing and integrating a longitudinal PRO framework into routine hospital data collection systems.

## Abbreviations

EORTC: European Organisation for Research and Treatment of Cancer
FACT-L: Functional assessment of cancer therapy-lung
PRO: Patient-reported outcomes
PROM: Patient reported outcome measures
PROMIS: Patient-Reported Outcomes Measurement Information System
QoL: Quality of life
